# Two-Sample Mendelian Randomization Study Identifies Tissue-Dependent Risk Genes in Autoimmune Diseases

**DOI:** 10.3390/cimb46110731

**Published:** 2024-10-31

**Authors:** Ryan Chiu, Li Ma

**Affiliations:** Department of Animal and Avian Sciences, University of Maryland, College Park, MD 20742, USA; chiur@bxscience.edu

**Keywords:** autoimmune disease, genomics, transcriptome, Mendelian randomization

## Abstract

Autoimmune diseases are among the most prevalent diseases across the world with genetic and environmental factors that contribute to their etiology. Because the exact causes of autoimmune diseases are largely unknown, a Mendelian randomization (MR) approach is used here to examine the potential causal association between gene expression levels and disease risk across various tissues. Specifically, this study focuses on six autoimmune diseases including Crohn’s disease, ulcerative colitis, rheumatoid arthritis, multiple sclerosis, type 1 diabetes mellitus, and systemic lupus erythematosus. Several of these diseases are currently treatable with immunosuppressants that target specific genes, such as *TNF-alpha*, *IL-23*, *CD20*, and more. In this study, a two-sample MR analysis is performed with multitissue expression quantitative trait loci (eQTLs) and large-scale genome-wide association studies to investigate how gene expression can influence the risk of developing these diseases. Our results show that genes *HLA-DQA1/2*, *HLA-DRB1/6*, *HLA-DQB2*, *C4A*, *CYP21A2*, and *HLA-DQB1-AS1* have a high causal effect across several diseases and tissues, and almost all of these findings originate from the major histocompatibility complex (MHC) region on Chromosome 6. Our findings support the current knowledge of genes associated with these diseases while also revealing novel genes that can be used for drug therapies in the future. Although several drug therapies currently exist to treat this selection of autoimmune diseases, we provide further insights into the main, common pathways responsible for autoimmune disease pathogenesis and discuss novel genes that lack research focus.

## 1. Introduction

Autoimmune diseases are caused by abnormal immune activity where the body attacks its own cells [1]. Approximately ten percent of the population has some form of autoimmunity, making it the third most common category of disease [2]. Autoimmune diseases are multifactorial and can be linked to various factors in a person’s genetics and environmental background. Genetic factors play a large role in the regulation of the proper function of the immune system and may lead to dysregulated immune system and disease conditions [3]. Although environmental conditions have also been found to play a big role in the risk of developing these diseases, we focus specifically on the genetic aspects. Autoimmune diseases vary greatly in pathogenesis, and we study six of the most common autoimmune diseases to provide an insight into the common and specific genetic factors, including Crohn’s disease (CD), ulcerative colitis (UC), rheumatoid arthritis (RA), multiple sclerosis (MS), type 1 diabetes mellitus (T1D), and systemic lupus erythematosus (SLE).

CD and UC are two forms of inflammatory bowel disease (IBD) [4]. While CD is generally localized in the area from the end of the small intestine (terminal ileum) to the start of the colon, it can still be found in any part of the gastrointestinal (GI) tract. UC, alternatively, can be seen anywhere in the colon and rectum. These two diseases have similar symptoms such as diarrhea, weight loss, abdominal pain, weight loss, and other related GI symptoms [4]. RA is another form of autoimmune disease that is often found in a person’s joints [5]. RA can attack multiple joints at once and is most commonly found at the joints of the hands, wrists, and knees, and is also known to affect other systems, such as the eyes, lungs, heart, and more. It is often associated with variants in the HLA complex and genes encoding cytokines and is especially prevalent in older women [6]. MS is considered as a continuum of inflammatory and neurodegenerative processes, which fluctuate and evolve over time and vary among individuals [7]. MS does not directly fit into the standard T-cell autoimmune dogma [8], and there is a debate on whether or not inflammation is the initial trigger of the disease or a secondary response [9]. Studies of common autoimmune diseases show that MS has a low rate of co-occurrence with other autoimmune diseases [2,10]. Myelin sheaths are the protective covering of nerve cells and help make the electrical impulses within a nerve cell more efficient, speeding up the signal. Because of this, it can result in cognitive impairment, reduced motor function, and weakness or numbness in limbs. T1D, unlike type 2 diabetes, where there is reduced insulin production or decreased insulin efficiency, is an autoimmune disease characterized by the destruction of the beta cells [11]. This leads to a deficiency in insulin which causes hyperglycemia, increased thirst, weight loss, fatigue, and extreme hunger. SLE is an autoimmune disease that, unlike the other diseases mentioned, causes inflammation in a multitude of tissues and organs, including the skin, joints, kidneys, cardiovascular system, respiratory system, brain, nervous system, and blood [12]. There are a wide range of symptoms including joint pain and swelling, skin rashes, chest pain and discomfort, and neurological symptoms. SLE also has a female-to-male ratio of 9:1 and can be traced back to variants of the HLA complex and cytokines [13].

Through the use of genome-wide association studies (GWAS), many genes have been identified to be correlated with the risk of autoimmune diseases, which need to be further verified by functional studies and clinical trial experiments [14]. GWAS studies have significantly advanced our understanding of autoimmune diseases, particularly highlighting the role of the HLA region and other non-HLA genes like *PTPN22* and *IL23R* [15,16,17]. While these studies have reported shared genetic factors across various autoimmune diseases, challenges remain including the “missing heritability” and functional interpretation of noncoding variants. Emerging approaches, including the integration of GWAS with multiomics data, the development of polygenic risk scores, and Mendelian randomization (MR) studies, aim to enhance the functional understanding and clinical applications of these findings.

Mendelian randomization studies can identify and analyze the causal effects of genetic variants on autoimmune diseases by integration of existing GWAS and functional genomics datasets [18]. MR simulates a randomized control trial, given the nature of randomly inherited alleles in the genome [19]. Single nucleotide polymorphisms (SNPs) are used as instrumental variables (IV) which are then calculated via a ratio of their effect on the exposure/outcome variable. Each SNP is associated with a specific phenotype so that the ratio calculated can represent the causal influence of a phenotype on the outcome. GWAS and MR have been increasingly integrated to investigate the causal relationship between phenotypes and disease outcomes. The addition of using transcriptome data (eQTL as IV) also allows for a better understanding of the biological mechanisms of diseases [20]. Moreover, gene expression is unique to different tissues given their unique functions, so they allow us to identify the association between specific tissues and diseases.

While GWAS and MR analyses have been used to study autoimmune diseases in previous studies [21,22], this study specifically focuses on gene expression levels to find out how gene regulation plays a role in autoimmune disease risk and how this differs across tissues. Specifically, we use eQTL data from the Genotype-Tissue Expression Consortium (GTEx, v8) and MR-Base to perform two-sample MR analysis [23,24]. Through the use of GWAS, MR, and eQTL, we investigate how the expression level of genes may influence the risk of developing autoimmune diseases. Moreover, we discuss any potential shared genetic dispositions and how they affect the pathogenesis of these diseases.

## 2. Materials and Methods

### 2.1. Mendelian Randomization (MR)

**Figure 1:**
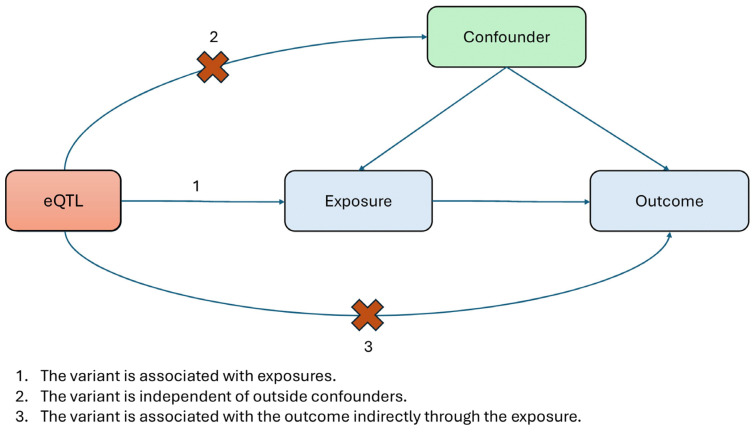
Overview of the Mendelian randomization analysis.

Mendelian randomization allows us to estimate a causal relationship between exposures and outcomes by evaluating the causal effect of an instrumental variable (IV) on both exposure and outcome [25]. Due to the mechanisms of genetic inheritance, any person’s alleles at any given gene are expected to be randomized. MR serves as a way to mimic randomized controlled trials to detect causal relationships by exploiting this natural process (Figure 1). This analysis framework is dependent on three assumptions: (1) the IV is associated with the exposure; (2) there are no confounding variables that influence the exposure effect on the outcome; and (3) the IV does not affect the outcome directly, but rather through the given exposure. In this study, we use gene expression levels as exposure and autoimmune disease risks as outcome. And genetic variants that influence gene expression levels (eQTLs) are IVs and tested against various autoimmune diseases to determine the causal effect of gene expression on disease risk. Our study focuses on transcriptome data and most genes have only a single eQTL SNP as IV, so the analyses are not available for thoroughly checking the assumptions, including horizontal pleiotropy.

### 2.2. Two-Sample MR (MR-Base)

To perform MR analysis, we use the ‘TwoSampleMR’ R Package (0.5.7, https://mrcieu.github.io/TwoSampleMR, accessed on 20 June 2024) and ‘MRInstruments’ R package (https://github.com/mrcieu/mrinstruments, accessed on 20 June 2024) from the MR-Base platform [23]. Two-sample MR allows datasets from different populations to be compared, is less prone to data overfitting compared to one-sample MR, and increases the power of our analysis. Through the MR-Base package, we harmonize eQTL data from GTEx (v8) with GWAS summary statistics data from the NHGRI-EBI GWAS Catalog [26]. The GWAS datasets (Table 1) are from these GWAS IDs (CD: ukb-b-8210, T1D: ebi-a-GCST90014023, Lupus: ebi-a-GCST90018917, RA: ebi-a-GCST90038685, MS: ukb-b-17670, UC: ebi-a-GCST90038684). GTEx data are publicly available and downloaded from the MRInstruments R package. Due to data availability, we focus predominantly on individuals of European ancestry.

### 2.3. Harmonizing and Data Cleaning

Harmonizing is the process of combining the summary-level statistics datasets from both the exposure and outcome studies and is required to determine any causal effect in MR. This is performed by ensuring that the effect allele, along with its corresponding effect size (beta) and standard error (se) values, from the outcome dataset matches the same effect allele from the exposure dataset. One potential problem in two-sample MR is the presence of palindromic SNPs. These occur when the alleles of an SNP correspond to the nucleotides that pair with each other. For example, if the effect allele is sequenced as A and the other allele is sequenced as T, then it becomes very difficult to determine which allele is the effect allele. SNPs can be sequenced on either the forward strand (5′ to 3′) or reverse strand (3′ to 5′), which may result in the same SNP being reported as its complementary base pairs (A may be reported as T if sequenced from the reverse strand) [23]. While the effect allele can easily be determined in nonpalindromic SNPs, palindromic SNPs additionally require that effect allele frequencies are reported and that the minor allele (rarer genotype) frequency is significantly lower. For these reasons, we remove all palindromic SNPs from this analysis. Additionally, we limit our exposure data to only cis-eQTLs and remove trans-eQTL SNPs as cis-eQTLs have stronger effect and are less likely to violate MR assumptions. Cis-eQTLs refer to genetic variants that affect local gene expressions within the same haplotype, while trans-eQTLs refer to variants that are on different chromosomes or located far away from the regulated genes on the same chromosome [27]. The threshold for eQTL inclusion is a *p*-value threshold of <1.0  ×  10^−5^, knowing that our analyses are confined to the top eQTL only. This threshold is used to maximize the number of possible genes analyzed across tissues.

### 2.4. Statistical Tests

A Wald ratio test estimates a causal effect by dividing the beta value (measurement of the influence of an SNP) for the outcome by the beta value for the exposure. A high Wald ratio means that the data point is significantly different from the hypothesized value and that the null hypothesis can be rejected. This is indicative of vertical pleiotropy and that the exposure has an effect on the outcome. Vertical pleiotropy can estimate a high causal effect while horizontal pleiotropy, where the SNP has a higher influence on the outcome than the exposure, would violate the assumption that the SNP does not affect the outcome. Because of this, we focus on the gene variants that show a high Wald ratio and low *p*-value.

Alternatively, the inverse variance-weighted (IVW) method allows us to analyze gene expression levels with multiple eQTL SNPs. This method finds the Wald ratio for each SNP and weights them based on the inverse of the variance of association between the exposure and outcome [28]. Wald ratios that have higher variance are weighted lower than those with lower variance, thus giving a more precise estimate of the causal effect of the exposure on the outcome.

We use a Wald ratio test for eQTLs with single SNPs as instruments or an inverse variance-weighted (IVW) method for instruments with more than one SNP in MR-Base to test the causal effect between exposure and outcome. To account for multiple testing, for each disease, the *p*-value threshold was calculated by dividing 0.05 by the number of tests of the respective dataset after filtering out palindromic and trans-eQTL SNPs.

## 3. Results

To present the results, we summarize in the Supplemental Tables all the significant gene–tissue pairs that passed the multiple-testing corrected significance threshold. Furthermore, we organize the data to visualize the frequency of significant genes among multiple tissues, the average and median of *p*-values, and effect size (beta) values to show the magnitude of effect size. Because of our large datasets, we use the median *p*-value to evaluate the most statistically significant genes, so a large *p*-value does not disproportionately bias the results. We also do this for the various tissues to find out what tissues play a more significant role than others. Afterward, we display the statistically significant genes we find on a Manhattan plot to show their chromosomal locations. For each disease, we highlight genes that had exceptionally low *p*-values, large beta values, or genes that were notably present across several diseases (Supplemental Tables).

### 3.1. Crohn’s Disease (CD)

**Figure 2:**
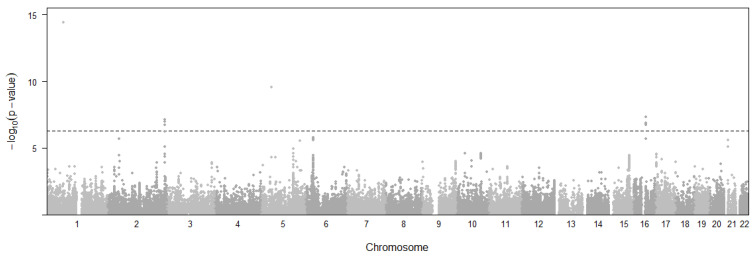
Manhattan plot representing the distribution of eQTL variants across chromosomal location and significance level for Crohn’s disease. Horizontal dashed line represents the *p*-value threshold for significant SNPs.

Ten eQTL variants and six genes (Figure 2) were found to be associated with CD risk with *p*-values below a threshold of *p* < 5.33×10^−7^. The threshold was decided with 0.05/n (n = 93,744) using a Bonferroni approach. Gene *IL12RB2* expression in esophagus mucosa (*p* = 3.82 × 10^−15^, β = −0.004) is found to have the lowest *p*-value, while the *AT16L1* (*p* = 7.43 × 10^−8^, Brain Cerebellum; *p* = 1.08 × 10^−7^, Testis), *NOD2* (*p* = 1.35 × 10^−7^, Skin; *p* = 1.60 × 10^−7^, Esophagus Mucosa), and *CYLD* (*p* = 1.35 × 10^−7^, Skin; *p* = 1.77 × 10^−7^, Esophagus Mucosa) genes are notable for having statistically significant associations in multiple tissues. More importantly, Esophagus Mucosa is the most frequent tissue for CD involving three genes, which is known to be relevant for disease pathogenesis [29].

### 3.2. Ulcerative Colitis (UC)

**Figure 3:**
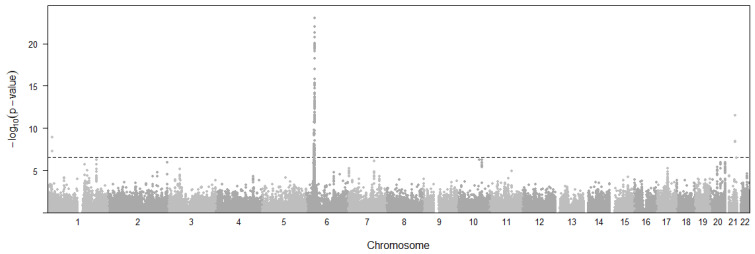
Manhattan plot representing the distribution of eQTL variants across chromosomal location and significance levels for ulcerative colitis. Horizontal dashed line represents the *p*-value threshold for significant SNPs.

A total of 196 eQTL variants of 57 genes (Figure 3) were found to be associated with UC with *p*-values below a threshold of *p* < 2.49 × 10^−7^. The threshold was decided with 0.05/n (n = 200,977). Most of these genes are located in or near the human major histocompatibility complex (MHC) region on Chromosome 6 (Figure 3). Among all significant gene–tissue pairs, *HLA-DRB1* (*p* = 4.72 × 10^−20^ across 23 tissues), *HLA-DQA1* (median *p* = 5.59 × 10^−14^ across 26 tissues), *HLA-DQB2* (*p* = 6.15 × 10^−14^ across 16 tissues), and *HLA-DQA2* (*p* = 2.64 × 10^−13^ across 26 tissues) showed the most significant pleiotropic associations with UC in more than 15 tissues. The results also show that Nerve Tibial is the most frequent tissue among the significant associations with UC.

### 3.3. Rheumatoid Arthritis (RA)

**Figure 4:**
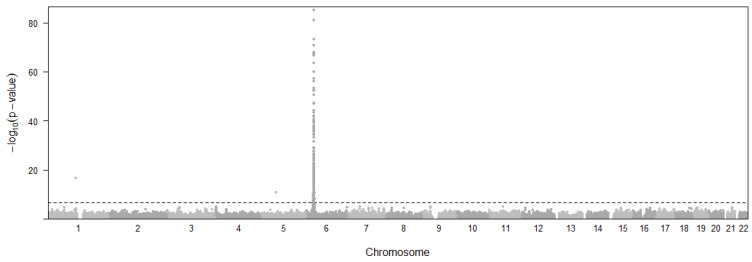
Manhattan plot representing the distribution of eQTL variants across chromosomal location and significance levels for rheumatoid arthritis. Horizontal dashed line represents the *p*-value threshold for significant SNPs.

A total of 528 variants of 117 genes (Figure 4) were found to be causally associated with RA with *p*-values below a threshold of *p* < 2.49 × 10^−7^. The threshold was decided with 0.05/n (n = 200,977). Similar to UC, the majority of significant genes are located in or near the human MHC region on Chromosome 6 (Figure 4). In all significant results, *HLA-DRB6* (median *p* = 2.61 × 10^−67^, 29 tissues), *HLA-DQA1* (median *p* = 2.68 × 10^−17^, 26 tissues), *HLA-DRB5* (median *p* = 3.49 × 10^−17^, 20 tissues), *HLA-DQA2* (median *p* = 2.3 × 10^−16^, 25 tissues), *SKIV2L* (median *p* = 1.8 × 10^−13^, 28 tissues), and *PSORS1C3* (median *p* = 1.06 × 10^−13^, 26 tissues) showed the most significant pleiotropic associations with RA in at least 20 tissues. Among the significant results, the most frequent tissues include Testis, Cells Transformed Fibroblasts, Skin, Lung Artery Tibial, and Adipose Subcutaneous.

### 3.4. Multiple Sclerosis (MS)

**Figure 5:**
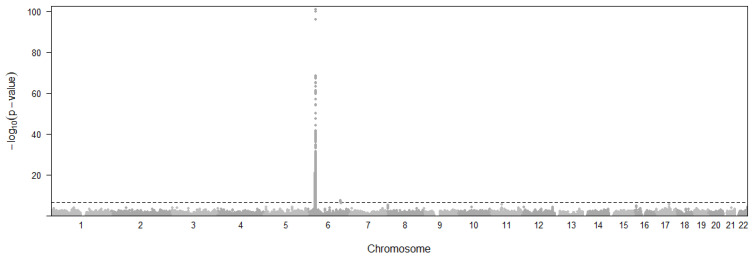
Manhattan plot representing the distribution of eQTL variants across chromosomal location and significance levels for multiple sclerosis. Horizontal dashed line represents the *p*-value threshold for significant SNPs.

A total of 563 variants of 145 genes (Figure 5) were found to be causally associated with MS with *p*-values below a threshold of *p* < 4.59 × 10^−7^. The threshold was decided with 0.05/n (n = 108,955). In all tissues, *HLA-DQB1* (median *p* = 1.99 × 10^−68^, 11 tissues), *HLA-DRB1* (median *p* = 7.08 × 10^−62^, 25 tissues), *HLA-DRB6* (median *p* = 9.54 × 10^−41^, 21 tissues), *CYP21A1P* (median 7.84 × 10^−29^, 14 tissues), *HLA-J* (median *p* = 2.82 × 10^−18^, 30 tissues), *HLA-DQA1* (median *p* = 1.53 × 10^−27^, 19 tissues), and *ATF6B* (median *p* = 3.1 × 10^−27^, 16 tissues) showed the most significant pleiotropic associations with MS. More importantly, the most frequent tissues are Brain Cerebellum, Esophagus Mucosa, Testis, Esophagus Muscularis, Nerve Tibial, Whole Blood, Colon Transverse, and Adipose Visceral Omentum. Among these, the Brain Cerebellum and Nerve Tibial are related to the central nervous system that is attacked in MS [30].

### 3.5. Type 1 Diabetes (T1D)

**Figure 6:**
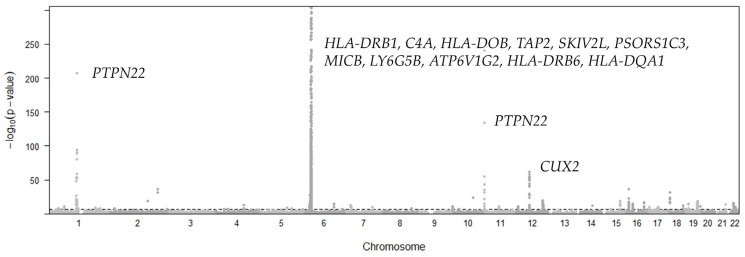
Manhattan plot representing the distribution of eQTL variants across chromosomal location and significance levels for Type 1 diabetes. Horizontal dashed line represents the *p*-value threshold for significant SNPs.

A total of 2940 eQTL variants of 508 genes (Figure 6) were found to be causally associated with T1D with *p*-values below a threshold of *p* < 4.59 × 10^−7^. The threshold was decided with 0.05/n (n = 108,955). The associated genes are distributed on several chromosomes in addition to a major peak in the MHC region. Due to the extremely small *p*-values from the original GWAS studies, our results are highly significant, and the smallest *p*-values were rounded to 1.53 × 10^−305^. In all tissues, *HLA-DRB1* (median 1.53 × 10^−305^, 21 tissues), *C4A* (median *p* = 6.91 × 10^−263^, 31 tissues), *HLA-DOB* (median *p* = 8.74 × 10^−232^, 27 tissues), *TAP2* (median *p* = 1.7 × 10^−231^, 23 tissues), *SKIV2L* (median *p* = 6.51 × 10^−148^, 28 tissues), *PSORS1C3* (median *p* = 1.8 × 10^−119^, 29 tissues), *MICB* (median *p* = 2.84 × 10^−88^, 28 tissues), *LY6G5B* (median *p* = 5.26 × 10^−81^, 28 tissues), *ATP6V1G2* (median *p* = 1.43 × 10^−68^, 21 tissues), *HLA-DRB6* (median *p* = 1.13 × 10^−65^, 29 tissues), *HLA-DQA1* (median *p* = 6.87 × 10^−63^, 26 tissues), *NOTCH4* (median *p* = 4.01 × 10^−62^, 24 tissues), and *HLA-DQB2* (median *p* = 3.2 × 10^−58^, 22 tissues) showed the most significant pleiotropic associations with T1D. The most frequent significant tissues include Testis, Skin, Adipose Subcutaneous, Thyroid, Whole Blood, Artery Tibial, and Nerve Tibial.

### 3.6. Systemic Lupus Erythematosus (SLE)

**Figure 7:**
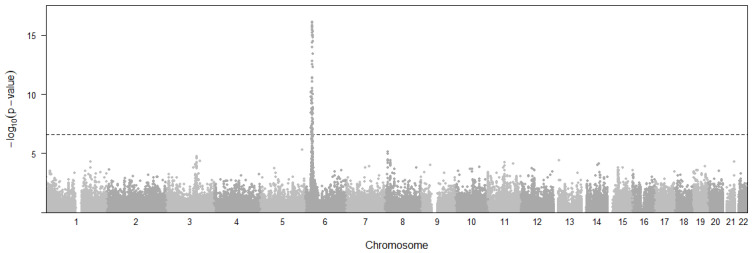
Manhattan plot representing the distribution of eQTL variants across chromosomal location and significance levels for systemic lupus erythematosus. Horizontal dashed line represents the *p*-value threshold for significant SNPs.

A total of 119 variants of 43 genes (Figure 7) were found to be causally associated with SLE with *p*-values below a threshold of *p* < 4.59 × 10^−7^. The threshold was decided with 0.05/n (n = 108,955). In all tissues, the *C4A* (median *p* = 1.2 × 10^−15^, 28 tissues), *CYP21A1P* (median 3.3 × 10^−15^, 12 tissues), and *C4B* (median *p* = 2.7 × 10^−15^, 6 tissues) genes were the top pleiotropic associations with SLE. The most frequent significant tissues are Nerve Tibial, Whole Blood, Pancreas, Adrenal Gland, Artery Aorta, Thyroid, and Muscle Skeletal.

### 3.7. Shared Risk Genes Across Multiple Autoimmune Diseases and Related Tissues

The MHC region showed the most significant signal for five out of six autoimmune diseases in this study, except for Crohn’s disease. The MHC region is still one of the major association signals for Crohn’s disease. Many genes within the MHC region are related to multiple diseases in multiple tissues with the top ones shown in Table 2. The *HLA-DQA1* gene has been previously associated with several autoimmune diseases [31,32], and is related to UC, RA, MS, and T1D in this study across many tissues. The *HLA-DQA2* gene has been associated with RA previously [33], and is related to UC, RA, MS, and T1D in many tissues in this study. The *CYP21A1P* gene has been associated with all five autoimmune diseases with evidence from multiple tissues.

## 4. Discussion

While many studies have explored the effect of various environmental factors as exposures for autoimmune diseases, this study focuses on the use of gene expression levels in conjunction with Mendelian randomization to identify many genes involved in the pathogenesis of several common autoimmune diseases. Across all tissues and six autoimmune diseases, we find that type 1 diabetes mellitus, rheumatoid arthritis, multiple sclerosis, and ulcerative colitis share several common genetic factors that may lead to increased risk of each other. Almost all of the identified genes were on the sixth chromosome across all diseases. Within this chromosome, *HLA-DQA1*, *HLA-DRB6*, *HLA-DQA2*, *HLA-DQB2*, *HLA-DRB1*, and *HLA-DQB1-AS1* were notable for having causal associations among several autoimmune diseases. This selection of genes is located in the MHC class II region. The MHC region is one of the largest collections of immune regulation genes. Because of this, genetic factors associated with the development of autoimmunity can be traced back to this region. Specifically, this region is among the most polymorphic within the genome and contains extreme linkage disequilibrium, meaning that alleles in this region are often inherited together, allowing for a diverse range of genetic variations across ethnicities [34]. While this diversity allows for a strong adaptive immune system, this also leads to certain alleles being associated with autoimmunity. While some genes identified in this study, such as *SKIV2L*, exist outside of the MHC region, they are few and suggest other independent risk factors for autoimmunity. Although MS is found to have a low rate of co-occurrence with other autoimmune diseases [2,3], the present study shows that MS share several common genetic factors that may lead to increased risk of each other.

In our results, we also find a variety of pseudogenes (*HLA-DRB6*, *CYP21A1P*, *PSORS1C3*) that have a significant effect on the risk of autoimmune diseases. Pseudogenes are imperfect copies of functional genes that cannot translate into a protein. They are structurally very similar to their parent genes, but often lack exons and introns, or promoters and stop codons crucial to code for proteins. These pseudogenes are believed to exist for various reasons, such as accumulated mutations that render genes dysfunctional due to a lack of selection pressure [35]. Although pseudogenes are unable to properly translate into proteins, some are still able to transcribe into ncRNA (noncoding RNA). While ncRNA cannot code for a protein, it has been found to have an effect on the regulation of gene expression by influencing epigenetics. This occurs by modifying histones, affecting heterochromatin formations, and DNA methylation [36]. Because this study focuses specifically on eQTLs as IVs for the exposure variable, we can find results related to pseudogenes that may not otherwise be found in other types of studies. Furthermore, this paper reveals that factors beyond mutations in protein-coding genes play a significant role in the risk of autoimmune disease and can widen the scope for early diagnosis based on family history. These pseudogenes may also reveal additional information about the evolutionary history of autoimmune diseases. As of now, pseudogenes also serve another important purpose of recording ancient genes that have lost their purpose. These can be used to discover the rate of gene duplication and are extremely helpful in phylogenetic studies. With our results of pseudogenes that play a significant role in regulating disease risk, future studies can trace some autoimmune diseases to ancient selective pressures and discover the origin of autoimmunity.

Of course, our study presents limitations. The studies chosen as outcomes for our MR analysis were chosen based on the number of SNPs and sample size to maximize the power of the study and find as many significant genes as possible. However, it is evident that some studies lacked significant SNPs that were able to be harmonized with GTEx data, such as the Crohn’s disease results, which led to underwhelming results compared to the other diseases. Additionally, this study only used SNP data from European ancestry, which limits our ability to apply these results to other ethnicities and geographical regions, given that the MHC region is highly polymorphic and specialized. This is especially prominent in different ethnicities. Lastly, the lack of testing for linkage disequilibrium undermines some of the results as alleles may be inherited together and invalidates the assumption that all genes are inherited randomly and independently.

## 5. Conclusions

In conclusion, the use of two-sample MR and eQTL data allows for the discovery of the causal association between gene expressions and autoimmunity. Additionally, some autoimmune diseases were found to share certain genetic predispositions that may increase the risk of inheriting or developing other immune-related conditions, in particular those located in or near the MHC region.

## Figures and Tables

**Table 1:** Summary information of GWAS datasets of six diseases.

GWAS Study ID	Disease	Population	Sample Size	Number of SNPs
ukb-b-8210	CD	European	462,933	9,851,867
ebi-a-GCST90014023	T1D	European	520,580	59,999,551
ebi-a-GCST90018917	Lupus	European	482,911	24,198,877
ebi-a-GCST90038685	RA	European	484,598	9,587,836
ukb-b-17670	MS	European	462,933	9,851,867
ebi-a-GCST90038684	UC	European	484,598	9,587,836

**Table 2:** Top genes associated with multiple autoimmune diseases in multiple tissues.

Gene	Disease				
	Ulcerative Colitis	Rheumatoid Arthritis	Multiple Sclerosis	Type 1 Diabetes	Systemic Lupus Erythematosus
*HLA-DQA1*	26 tissues	26 tissues	19 tissues	26 tissues	
*HLA-DQA2*	26 tissues	25 tissues	10 tissues	27 tissues	
*HLA-DRB6*	2 tissues	29 tissues	21 tissues	29 tissues	
*HLA-DRB1*	23 tissues	6 tissues	25 tissues	21 tissues	
*HLA-DQB2*	16 tissues	18 tissues	12 tissues	22 tissues	
*C4A*	1 tissue	1 tissue		31 tissues	28 tissues
*CYP21A1P*	1 tissue	13 tissues	14 tissues	19 tissues	12 tissues
*HLA-DQB1-AS1*	11 tissues	18 tissues	3 tissues	18 tissues	

## Data Availability

GWAS datasets are downloaded from the EU GWAS catalog (https://gwas.mrcieu.ac.uk/, accessed on 20 June 2024) with GWAS IDs (CD: ukb-b-8210, T1D: ebi-a-GCST90014023, Lupus: ebi-a-GCST90018917, RA: ebi-a-GCST90038685, MS: ukb-b-17670, UC: ebi-a-GCST90038684). GTEx data are publicly available (https://gtexportal.org, accessed on 20 June 2024) and downloaded from the MR-Base program.

## Supplementary Materials

The following supporting information can be downloaded at: https://www.mdpi.com/article/10.3390/cimb46110731/s1, Table S1: Results of MR analysis for all six diseases. Table S2: Results of MR analysis for Lupus. Table S3: Results of MR analysis for MS. Table S4: Results of MR analysis for CD. Table S5: Results of MR analysis for T1D. Table S6: Results of MR analysis for RA. Table S7: Results of MR analysis for UC.
